# Diurnal variation of choriocapillaris vessel flow density in normal subjects measured using optical coherence tomography angiography

**DOI:** 10.1186/s40942-018-0140-0

**Published:** 2018-10-10

**Authors:** Salman Sarwar, Muhammad Hassan, Mohamed K. Soliman, Muhammad Sohail Halim, Mohammad Ali Sadiq, Rubbia Afridi, Aniruddha Agarwal, Diana V. Do, Quan Dong Nguyen, Yasir J. Sepah

**Affiliations:** 1 Mason Eye Institute, Missouri University, Columbia, MO USA; 2 Department of Ophthalmology, Byers Eye Institute, Stanford University, 2370 Watson Court, Palo Alto, CA 94303 USA; 3 Ocular Imaging Research and Reading Center, Menlo Park, CA USA; 4 Postgraduate Institute of Medical Education and Research, Chandigarh, India

**Keywords:** Optical coherence tomography angiography, Choroidal thickness, Choroidal vessel density, Optical coherence tomography

## Abstract

**Background:**

Vessel flow density (VFD) may provide important information regarding perfusion status. Diurnal variation in VFD of choriocapillaris has not been reported in literature. In the index study, optical coherence tomography angiography (OCTA) was used to assess the diurnal variation of the VFD in the choriocapillaris of subjects with no known ocular disease.

**Methods:**

Fifty eyes with no known ocular disease (25 subjects) were included. OCTA images were acquired using AngioVue (Optovue, Fremont, CA, USA) at two different time points on a single day: 9:00 AM and 6:00 PM. Macular cube scan protocol (3 × 3 mm) centered on the fovea was used. Automatic segmentation of the retinal layers and choriocapillaris was performed using ReVue software, which was also used to measure the choriocapillaris VFD. Horizontal line scan passing through fovea was obtained by the device at both time points to measure the subfoveal choroidal thickness (CT). Linear measurement tool of software was used to measure subfoveal CT according to a standardized reproducible method. Wilcoxon signed-rank test was used to assess the differences in choriocapillaris VFD and subfoveal CT at the two time points. Correlation between change in choriocapillaris VFD and subfoveal CT at the two time points was assessed using the Pearson correlation coefficient (r).

**Results:**

The mean age of the subjects was 31.96 ± 11.23 years. Choriocapillaris VFD was significantly higher at 9:00 AM compared to 6:00 PM (P < 0.0001) with mean choriocapillaris VFD of 68.74 ± 4.80% at 9:00 AM and 67.57 ± 5.41% at 6:00 PM, with a mean diurnal amplitude of 1.17%. Mean subfoveal CT was 287.74 ± 61.51 µm at 9:00 AM and 270.06 ± 60.73 µm at 6:00 PM. Subfoveal CT was also significantly higher at 9:00 AM compared to 6:00 PM (P < 0.0001) with a mean diurnal amplitude of 17.68 µm. Change in choriocapillaris VFD correlated with change in subfoveal CT (r = 0.87, P < 0.001).

**Conclusion:**

OCTA demonstrated significant diurnal change in choriocapillaris VFD in subjects without any ocular disease with VFD being higher in the morning and lower in the evening. Decrease in choriocapillaris VFD in the evening correlated with a reduction in subfoveal CT.

## Introduction

The choroid, a fully vascularized structure, is supplied by the posterior ciliary arteries, which branch from the ophthalmic artery and account for 85% of the total ocular blood flow [1]. High resolution optical coherence tomography has enabled in vivo imaging of choroid with unprecedented detail which was otherwise not possible [2]. Choroidal vessels are poorly autoregulated and changes in perfusion pressure directly affects the blood flow [3].

Choroid being primarily a microvascular structure has certain characteristics which makes it challenging to study clinically. Fenestration the capillaries of choriocapillaris results in dye leakage during fluorescein angiography (FA) and indocyanine green (ICGA) angiography thus making high resolution choriocapillaris imaging extremely challenging [4]. Optical coherence tomography angiography (OCTA) is a noninvasive imaging modality that can provide high resolution *en face* images of the retinal microvasculature and choriocapillaris with the use of split-spectrum amplitude decorrelation angiography algorithms (SSADA) [5, 6]. Though thickness of the choroidal vascular layer can be obtained using a variety of OCT techniques, measurement of choriocapillaris vessel flow density (VFD) in vivo has not been possible until the advent of OCTA.

Circadian changes in sub-foveal choroidal thickness in normal healthy subjects using spectral domain optical coherence tomography has previously been demonstrated. These studies have shown a diurnal rhythm with higher choroidal thickness in the morning time compared to evening [7, 8]. Choriocapillaris VFD can be obtained by using OCTA and may provide important information regarding perfusion status. Diurnal variation in VFD of choriocapillaris has not been reported in literature. In the index study, OCTA was used to assess the diurnal variation of the VFD in the choriocapillaris of subjects with no known ocular disease.

## Methods

This study employed a cross-sectional design. The study was approved by the local institutional review board and was conducted in compliance with the Declaration of Helsinki, the United States Code of Federal Regulations Title 21, and the Harmonized Tripartite Guidelines for Good Clinical Practice (1996). All subjects received detailed information about the nature of the investigation and provided written informed consent.

### Study population

The study included a cohort of normal subjects recruited from the ophthalmology clinics at a tertiary care eye center. Inclusion criteria for the study consisted of healthy volunteers with no known systemic or ocular diseases, except refractive error. Twenty eyes of 10 normal adults were included in the study.

### Optical coherence tomography angiography (OCTA)

Imaging was performed on all subjects using AngioVue OCTA system (Optovue, Fremont, CA, USA) by a single operator. The device has a high acquisition speed of 70,000 A-scans per second. All subjects were seated and properly aligned. After achieving stabilization with a forehead support and chinrest, he or she was instructed to focus on a fixation target during image acquisition. Each subject underwent an imaging session at 9:00 AM and 6:00 PM on a single day using the same imaging protocol. A single operator performed all the imaging sessions. The operator visualized the fovea by using the real-time *en fac*e view. Each imaging session included a macular cube scan over 3 × 3-mm region centered on the fovea. The device acquires two OCTA volume scans for each macular cube scan to decrease motion artifacts and fixation changes.

Computation of flow map for each scan was performed using SSADA algorithm. The algorithm distinguishes blood flow from static tissue by differentiating the rapid variation over time in signals from flowing blood cells compared to static tissue. The decorrelation algorithm is hence capable of identifying blood flow and generating perfusion maps with high resolution. Ocular movement during scan acquisition also generates decorrelation which are. The Optovue SSADA algorithm uses standardized techniques to account for motion artifacts allowing creation of motion-corrected flow data volumes. However, significant motion artifacts and poor signal strength may result in image lines and gaps when a perfusion map is created. OCTA scans were repeated in the same session if the OCTA image was noted to have gaps or lines. Only images of high quality were accepted to ensure standardized analysis.

ReVue software (Version: 2015.1.0.71) used by the OCTA systems performs automatic segmentation of the retinal layers and choriocapillaris. Three layers are obtained with the 3x3 mm macular cube scan: the superficial retinal capillaries, deep retinal capillaries, and the choriocapillaris. In this study, we included only the choriocapillaris layer for analysis.

### Choriocapillaris vessel flow density (VFD)

**Fig. 1:**
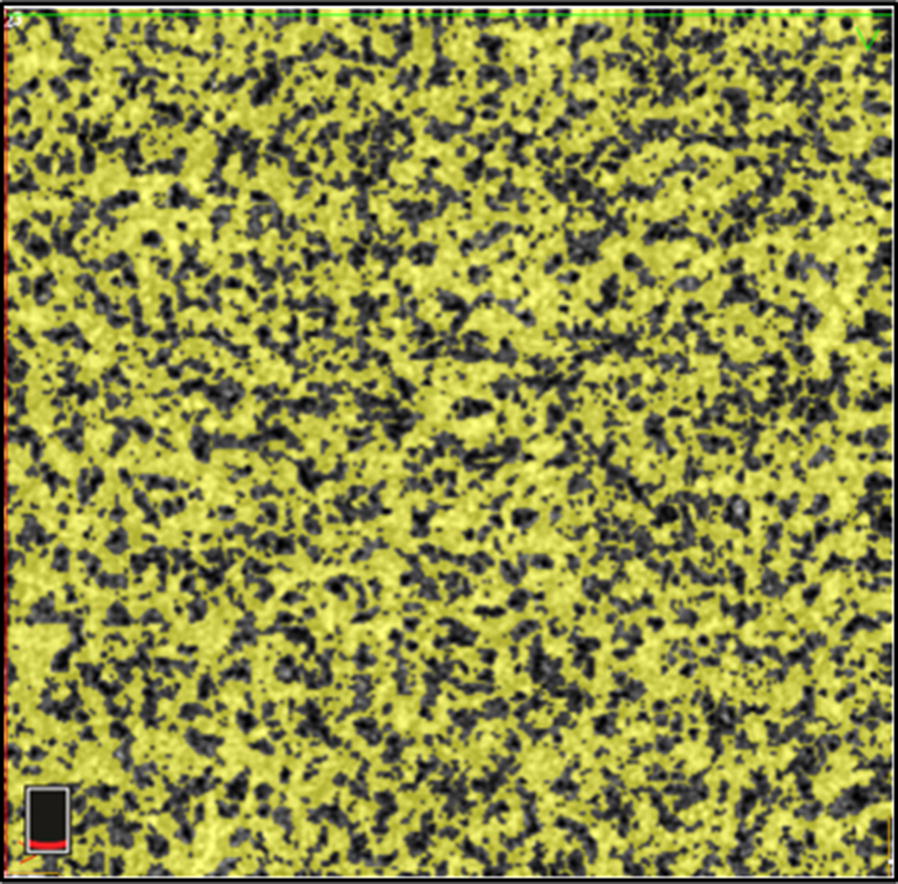
OCTA at the level of the choriocapillaris demonstrates vascular flow in the foveal region of a subject with no known ocular pathology. The yellow area depicts flow of the RBCs within the choriocapillaris

Measurement of the choriocapillaris VFD was performed by using ReVue software (Version: 2015.1.0.71) by an independent grader. The flow measurement tool of the software was used at the choriocapillaris level to measure the VFD (Fig. 1).

### Measurement of subfoveal choroidal thickness (CT)

**Fig. 2:**
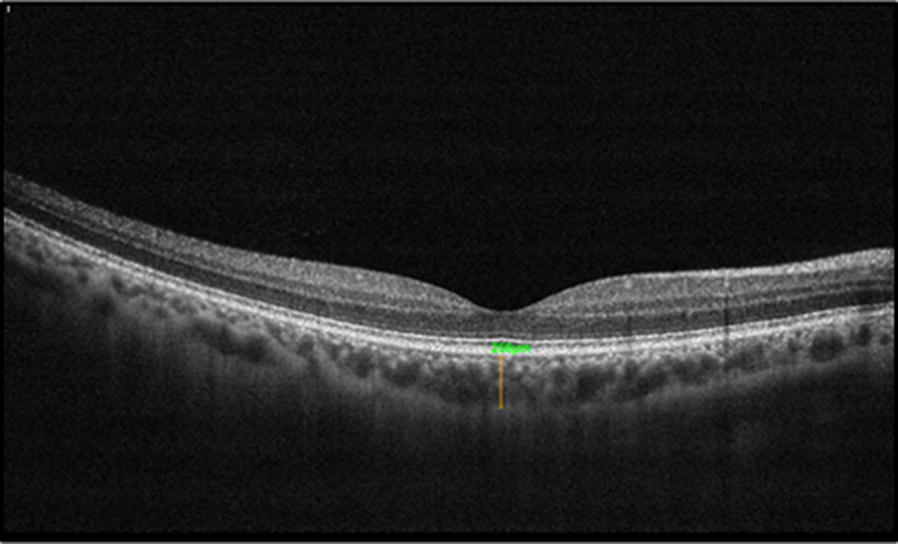
Horizontal line scan passing through fovea of a subject with no know ocular pathology. Subfoveal choroidal thickness measured using ReVue linear measurement tool

Using the enhanced depth imaging mode (EDI) of the AngioVue (Optovue, Fremont, CA, USA) device a horizontal line scan passing through the fovea was obtained on each eye during the imaging sessions at 9:00 AM and 6:00 PM. The EDI line scan was obtained immediately after the OCTA scan. ReVue linear measurement tool was used to measure subfoveal CT perpendicularly from the outer edge of the hyper-reflective retinal pigment epithelium to the inner sclera according to a standardized reproducible method (Fig. 2). Manual measurements of the choroidal thickness are highly reliable and reproducible.

### Statistical analysis

Wilcoxon signed-rank test was used to assess the difference in choriocapillaris VFD and subfoveal CT at the two time points. Correlation between change in choriocapillaris VFD and subfoveal CT at the two time points was assessed using the Pearson correlation coefficient (r). For all tests, values of P < 0.05 were considered statistically significant. Statistical analyses were performed using MedCalc for Windows, version 12.5.0.0 (MedCalc Software, Ostend, Belgium).

## Results

A total of 50 eyes of 25 normal subjects were included in the analysis. Table 1 outlines the demographic characteristics of the study population with no known ocular and systemic illnesses except refractive errors.

**Table 1:** Demographic characteristics

Mean age (SD) (years)	31.96 (11.23)
Gender (% male)	56
Race (%)	
Asian (n)	56 (14)
Caucasians (n)	40 (10)
African (n)	4 (1)
Mean BCVA (Snellen’s equivalent)	20/20
Mean central retinal thickness (µm) (SD)	252.8 µm (26.2)

**Fig. 3:**
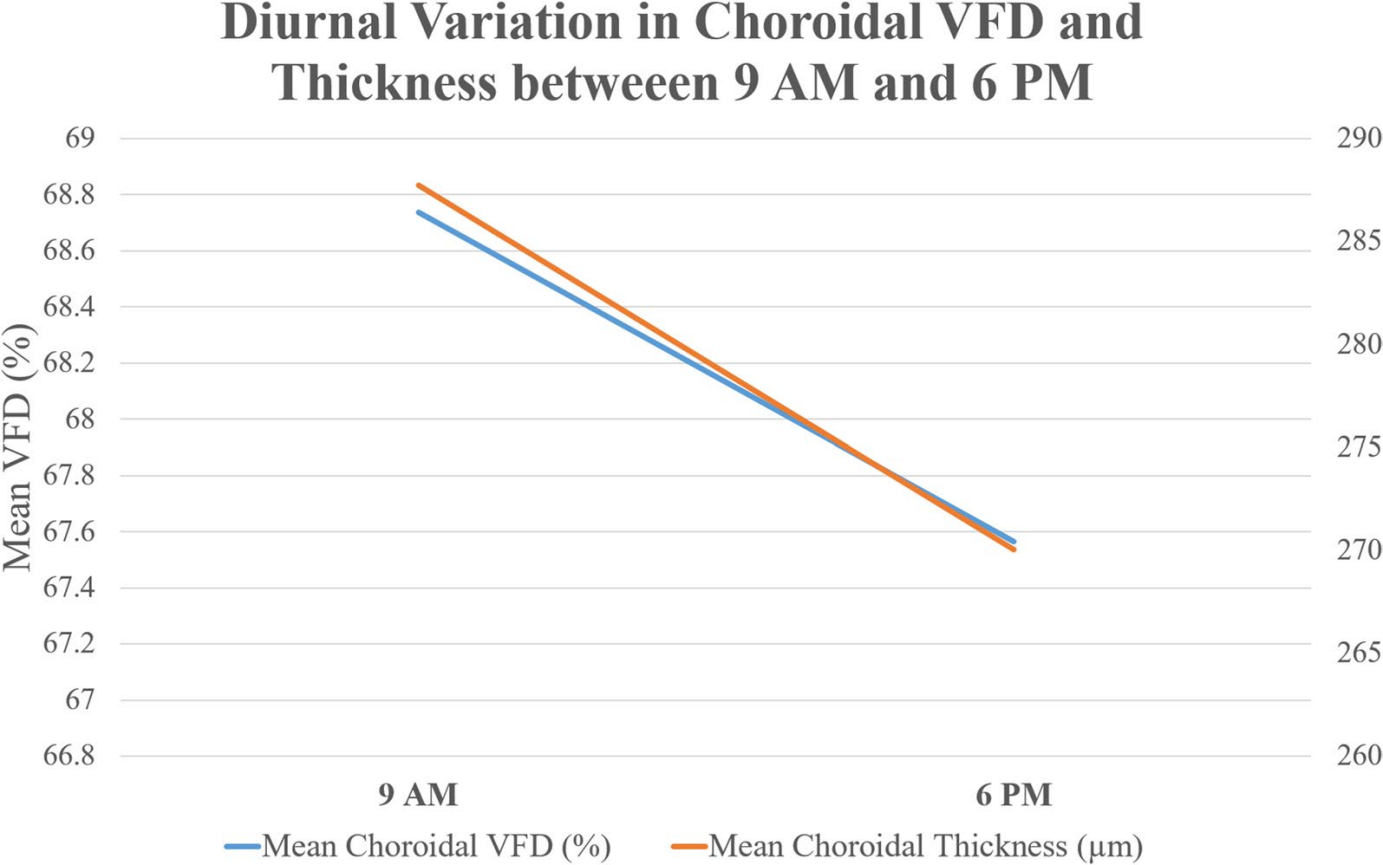
Line graph demonstrating diurnal variation in choroidal vessel flow density (VFD) and choroidal thickness between 9 AM and 6 PM

**Fig. 4:**
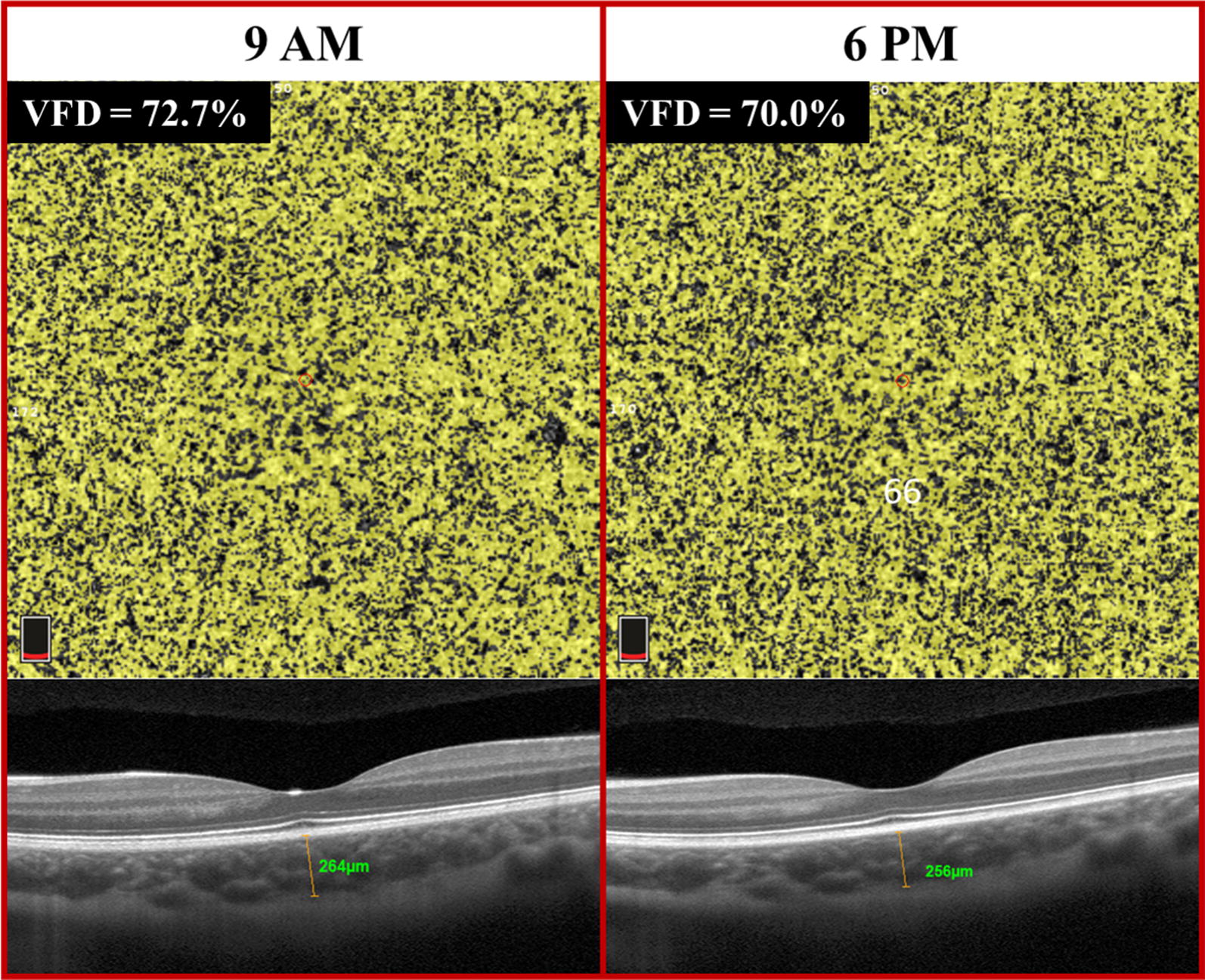
Choroidal vessel flow density (VFD) and choroidal thickness images of the a 21 year old Caucasian female demonstrating the variation in the choroidal VFD and thickness between the morning and evening

Mean choriocapillaris VFD at 9:00 AM was 68.74 ± 4.80%. At 6:00 PM, mean choriocapillaris VFD was 67.57 ± 5.41% (Figs. 3, 4). The mean diurnal amplitude was 1.17%. Choriocapillaris VFD was significantly higher at 9:00 AM compared to 6:00 PM (P < 0.0001).

Mean subfoveal CT was 287.74 ± 61.51 µm and 270.06 ± 60.73 µm at 9:00 AM and 6:00 PM, respectively (Figs. 3 4). Mean subfoveal CT was also significantly higher at 9:00 AM compared to 6:00 PM (P < 0.0001) with a mean diurnal amplitude of 17.68 µm.

Diurnal change in choriocapillaris VFD correlated with the diurnal change in subfoveal CT (r = 0.87, P < 0.001).

## Discussion

In the index study, OCTA demonstrated significant diurnal change in choriocapillaris VFD in subjects without any ocular disease. We observed a high VFD in the morning and lower in the evening. In addition, all subjects were observed to have a higher subfoveal CT in the morning. However, decrease in choriocapillaris VFD in the evening did not correlate with a reduction in subfoveal CT. Change in the diurnal amplitude of choriocapillaris VFD was statistically significant.

Previous investigations have demonstrated diurnal variation in subfoveal choroidal thickness and in other ocular parameters such as anterior chamber depth [9], axial length [9, 10] and intraocular pressure [11]. Although earlier studies have shown circadian changes in choroidal thickness but to our knowledge this is the first study demonstrating circadian changes in choroidal blood flow as demonstrated by changes in VFD.

The finding of a variation in choriocapillaris VFD is significant as it indicates that measurement of VFD of the choriocapillaris should take into account the time of the day imaging is performed. We also found that the decrease in choriocapillaris VFD correlated with decrease in choroidal thickness. This can be explained by the fact that choroidal thickness is a strong determinant of the choroidal VFD as shown by Fujiwara et al. [12]. Our finding of diurnal changes in choridal thickness is consistent with previous studies. Tan et al. investigated the circadian changes in subfoveal choridal thickness and it relationship with circulatory factors. They found a significant circadian changes in subfoveal chroidal thickness with higher values at 9:00 AM and decreasing thickness at 5:00 PM [8]. Usui et al. [7] also observed a similar pattern in subfoveal choridal thickness with thicker choroid in the morning and thinner in the evening time (9:00 PM).

OCTA has been successfully used to provide both quantitative and qualitative information of different types of choroid neovascularization in age related macular degeneration [5, 13]. Compared to the current gold standards for retinal and choroidal angiographic gold standards (fluorescein angiography and indocyanine green angiography), OCTA is non-invasive and provides volumetric scans with the ability of specific depth micro-meter resolution automatic segmentation. Image acquisition is fast with accurate size and localization information regarding lesions in addition to providing structural and blood flow information. Disadvantages of OCTA are its limited field of view, inability to view leakage, increased potential for artifacts (blinks, movement, vessel ghosting), and inability to detect blood flow below the slowest detectable flow.

It has been suggested previously that fluctuations in the ocular blood flow could be an important factor explaining the mechanism of circadian changes in subfoveal choroidal thickness [14, 15]. Previous studies have demonstrated no significant correlation between choroidal thickness and the mean arterial pressure, heart rate, intraocular pressure, and mean ocular perfusion pressure [7]. However, Jung et al. [15] demonstrated increase in choroidal thickness after drop in systolic blood pressure following hemodialysis. We observed decrease in vessel flow density having a positive correlation with decrease in choroidal thickness. The fluctuation in choriocapillaris vessel flow density may be secondary to circadian changes in choriocapillaris thickness.

Limitation of our study include a small sample size and relatively young subject cohort. In addition, we did not analyze correlation of choriocapillaris VFD with systemic or ocular factors. We utilized the automatic segmentation feature of the Optovue review software for segmentation of choriocapillaris layer for measurement of VFD which includes a portion of the retinal pigment layer in the boundary of choriocapillaris. Future studies with a large sample size may be needed to validate the findings of this study. Additionally, manually correction of segmentation may allow more accurate assessment of the choriocapillaris layer VFD.

## Conclusion

OCTA is becoming a prominent noninvasive tool for imaging and quantifying the choroidal vasculature. However, it is important to account for diurnal changes in choroidal vasculature when assessing parameters such as choroidal thickness and VFD. Our study has demonstrated that the choroidal VFD similar to choroidal thickness shows significant diurnal variation.

## Abbreviations

CT: choroidal thickness
OCTA: optical coherence tomography angiography
VFD: vessel flow density
SSADA: split-spectrum amplitude decorrelation angiography
